# The effects of high glucose on tendon-derived stem cells: implications of the pathogenesis of diabetic tendon disorders

**DOI:** 10.18632/oncotarget.15418

**Published:** 2017-02-16

**Authors:** Yu-Cheng Lin, Ying-Juan Li, Yun-Feng Rui, Guang-Chun Dai, Liu Shi, Hong-Liang Xu, Ming Ni, Song Zhao, Hui Chen, Chen Wang, Gang Li, Gao-Jun Teng

**Affiliations:** ^1^ Department of Orthopaedics, Zhongda Hospital, School of Medicine, Southeast University, Nanjing, Jiangsu, China; ^2^ Department of Geriatrics, Zhongda Hospital, School of Medicine, Southeast University, Nanjing, China; ^3^ Department of Orthopaedics, Xishan Peoples Hospital, Wuxi, Jiangsu, China; ^4^ China Orthopedic Regenerative Medicine Group, China; ^5^ Department of Orthopaedics and Traumatology, Faculty of Medicine, The Chinese University of Hong Kong, Hong Kong, SAR, China; ^6^ Program of Stem Cell and Regeneration, School of Biomedical Science, and Li Ka Shing Institute of Health Sciences, Faculty of Medicine, The Chinese University of Hong Kong, Hong Kong, SAR, China; ^7^ Department of Orthopaedics, The General Hospital of Chinese Peoples Liberation Army, Beijing, China; ^8^ Department of Radiology, Jiangsu Key Laboratory of Molecular and Functional Imaging, Zhongda Hospital, School of Medicine, Southeast University, Nanjing, Jiangsu, China

**Keywords:** tendon-derived stem cells, high glucose, diabetic tendon disorders, pathogenesis, Pathology Section

## Abstract

Patients with diabetes are at great risk to suffer many musculoskeletal disorders, such as tendinopathy, tendon rupture and impaired tendon healing. However, the pathogenesis of these tendon disorders still remains unclear. In this study, we aimed to investigate the effects of high glucose on cell proliferation, cell apoptosis and tendon-related markers expression of tendon-derived stem cells (TDSCs) in vitro. These findings might provide new insights into the pathogenesis of diabetic tendon disorders. The cell proliferative ability and apoptosis rate of TDSCs in different groups were evaluated by MTT assay and Annexin V-FITC/PI staining assay. The mRNA expression of tendon-related markers (Scleraxis and Collagen I alpha 1 chain) were assessed by qRT-PCR. The protein expression of tendon-related markers (Tenomodulin and Collagen I) were measured by Western blotting. The proliferative ability of TDSCs treated with high glucose (15mM and 25mM) decreased significantly at day1, day3 and day5. The cell apoptosis of TDSCs increased significantly when they were cultured with high glucose for 48h in vitro. The gene expression of Scleraxis and Collagen I alpha 1 chain in TDSCs decreased significantly when they were treated with high glucose for 24h and 48h. The protein expression of Tenomodulin and Collagen I in TDSCs decreased significantly when they were treated with high glucose for 24h and 48h. High glucose could inhibit cell proliferation, induce cell apoptosis and suppress the tendon-related markers expression of TDSCs in vitro. These findings might account for some pathological mechanisms underlying the pathogenesis of diabetic tendon disorders.

## INTRODUCTION

Patients with diabetes, both type 1 and type 2 are at greater risk to suffer many musculoskeletal disorders, such as tendinopathy, limited joint mobility, tendon ruptures, adhesive capsulitis and impaired tendon healing ability than non-diabetic patients [1–6]. Recently, a strong evidence has been reported that diabetes is associated with higher risk of tendinopathy [5]. The tendon seems to be particularly sensitive to impair with degenerative changes in about 40% of diabetic patients [3]. Diabetic patients have a greater risk of tendinopathy and/or traumatic rupture in musculoskeletal tissues because of the altered structural properties in tendon [7]. For the impaired Achilles tendons, patients with diabetes have higher proportion of postoperative infection and weaker tendon healing ability [6]. However, the underlying cellular and molecular mechanisms of the pathogenesis of diabetic tendon disorders are still unknown.

While tendons are supposed to be mainly comprised of tenocytes and collagens, recent studies have demonstrated that tendons from human, mice, rats, and rabbits also contain stem cell populations [8–11]. These stem cell populations isolated from tendon tissues were termed as tendon-derived stem cells (TDSCs). Like other adult stem cells, TDSCs have stem cell characteristics, including clonogenicity, self-renewing ability and the multi-differentiation potential [10]. TDSCs could promote tendon repair and regeneration, and might maintain tendon homeostasis [12, 13]. The depletion of the stem cell pool and erroneous (non-tenogenic) differentiation of TDSCs might play an essential role in the pathogenesis of tendinopathy [14–16]. TDSCs might also be involved in the pathogenesis of diabetic tendon disorders [17].

A recent study showed that high plasma glucose levels might be a risk factor for rotator cuff tear [18]. Hyperglycemia produces a reduction in proteoglycans levels, which may contribute to the tendon pathology observed clinically in diabetes [19]. High glucose level alters the response of tendon cells to oxidative stress and high glucose increases the expression of MMP-9 and MMP-13 in tendon cells *in vitro* [20, 21]. Nevertheless, the effects of high glucose concentration on TDSCs have not been investigated. We hypothesized that high glucose could impair cell proliferation, induce cell apoptosis and alter tendon-related markers expression of TDSCs, which might be a potential cellular mechanism of the pathogenesis of diabetic tendon disorders. In this study, we aims to investigate the effects of different glucose concentrations (5.5mM, 15mM, and 25mM) on the proliferation, apoptosis and tendon-related markers expression of rat TDSCs *in vitro*. All these findings might provide some new insights into the pathogenesis of diabetic tendon disorders.

## RESULTS

### Inhibition of cell proliferation

**Figure 1:**
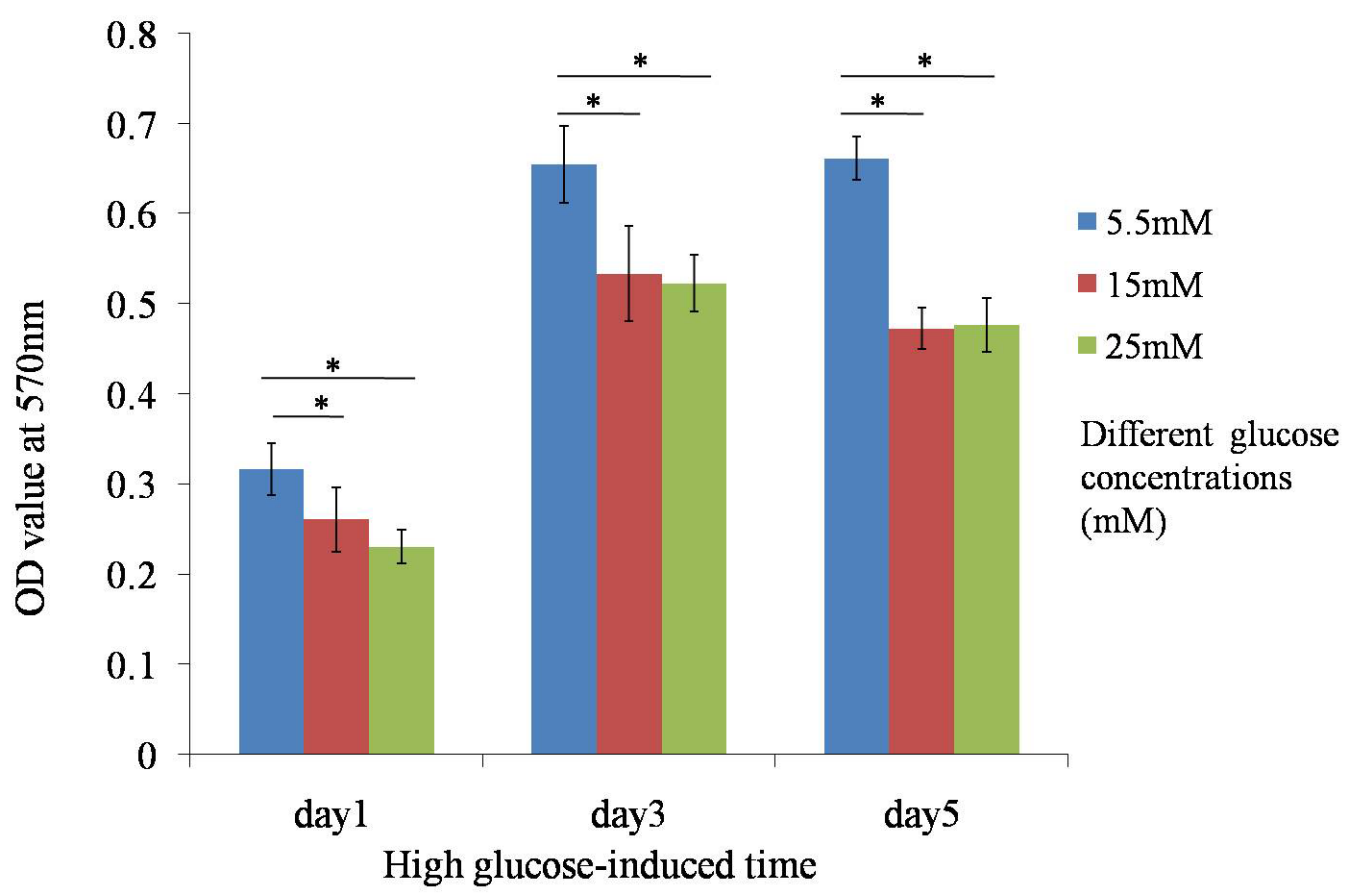
MTT assay Treatment of TDSCs with high glucose (15mM and 25mM) decreased cell proliferation at day1, day3 and day5. *P ≤ 0.05.

Cells were treated with different concentrations of glucose medium for 1 day, 3 days and 5 days, and viable cells were monitored by MTT assay. Cell morphology of TDSCs was slightly changed when they were treated with high glucose concentration for 24h. Treatment of TDSCs with high glucose (15mM and 25mM) decreased cell proliferation at day1, day3 and day5 (Figure 1). The results showed the significant differences between high glucose groups (15 and 25 mM) and normal glucose group (5.5mM), which implied that high glucose could inhibit TDSCs proliferation *in vitro*.

### Induction of cell apoptosis

**Figure 2:**
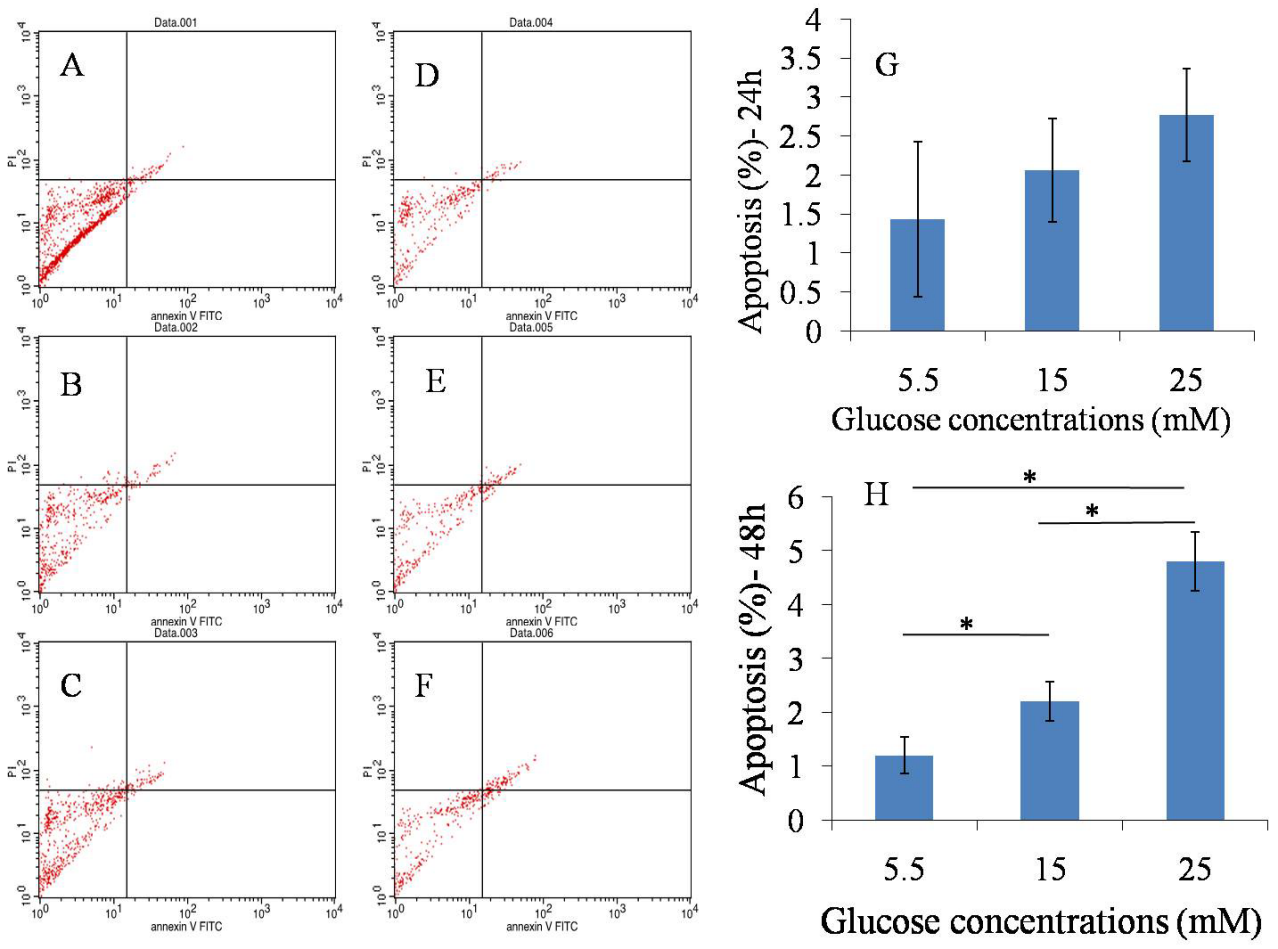
Apoptosis assay **A**., **B**., **C**.. 24h after 5.5mM, 15mM, 25mM glucose induction separately; **D**., **E**., **F**.. 48h after 5.5mM, 15mM, 25mM glucose induction separately; **G**. Statistical results in 24h; **H**. Statistical results in 48h.There was no statistical difference between high glucose groups (15 and 25 mM) and normal glucose group (5.5mM) after 24 hours culture, however, significant differences were observed between high glucose groups and normal glucose group at 48 hours. *P ≤ 0.05.

Viable, early and late apoptotic or necrotic cells can be identified by flow cytometry analysis using dual staining with annexin V/PI dyes. We treated TDSCs with different glucose concentrations (5.5mM, 15mM, and 25mM) for 24 hours and 48 hours, and measured the apoptosis rate. Flow cytometry analysis showed that there was no statistical difference between high glucose groups (15 and 25 mM) and normal glucose group (5.5mM) after 24 hours culture, however, significant differences were observed between high glucose groups (both 15 and 25 mM) and normal glucose group (5.5mM) at 48 hours (Figure 2). Overall, high glucose could induce cell apoptosis when TDSCs were cultured with high glucose for 48 hours *in vitro*.

### Suppression of the key tendon-related markers expression of TDSCs

**Figure 3:**
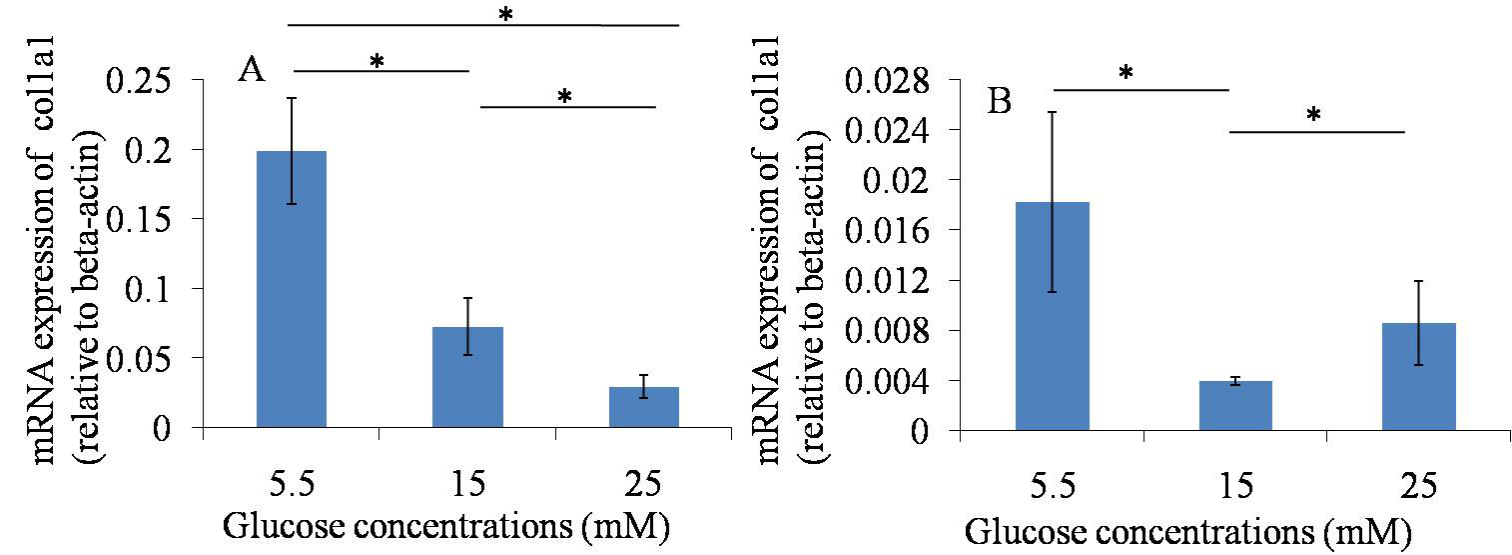
qRT-PCR assay----Col1a1 **A**. 24h after glucose induction; **B**. 48h after glucose induction; The mRNA expression of Col1a1 was down-regulated when TDSCs were treated with high glucose (15mM and 25mM) at 24 hours and 48 hours, compared with that in normal glucose group (5.5mM). *P ≤ 0.05 .

**Figure 4:**
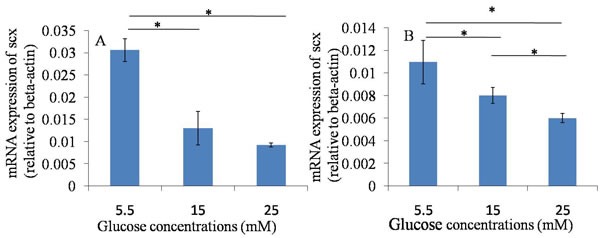
qRT-PCR assay----Scx **A**. 24h after glucose induction; **B**. 48h after glucose induction; The mRNA expression of Scx was down-regulated when TDSCs were treated with high glucose (15mM and 25mM) at 24 hours and 48 hours, compared with that in normal glucose group (5.5mM). *P ≤ 0.05.

The results of the qRT-PCR assay showed that the mRNA expression of Scx and Col1a1 were down-regulated when they were treated with 15 and 25 mM glucose at 24 hours and 48 hours, compared with those in normal glucose group (5.5mM). The results suggested that high glucose could down-regulate the mRNA expression of tendon-related markers of TDSCs after 24 hours and 48 hours culture. (Figures 3, 4).

**Figure 5:**
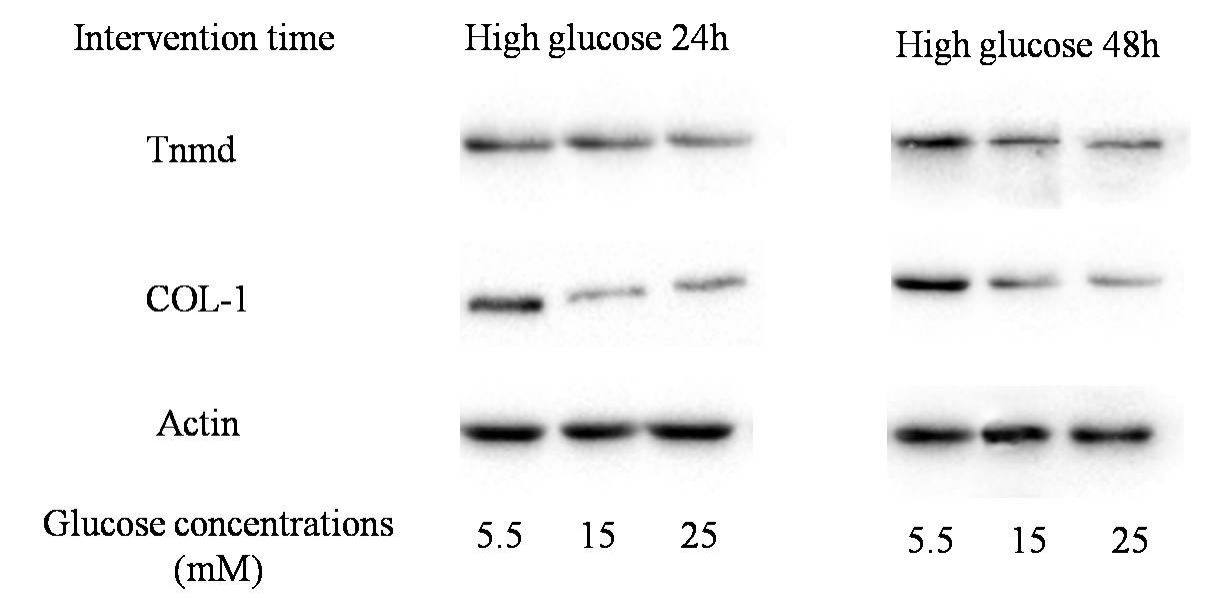
Western blotting assay The expression of tendon-related proteins (Tnmd and Col-1).

**Figure 6:**
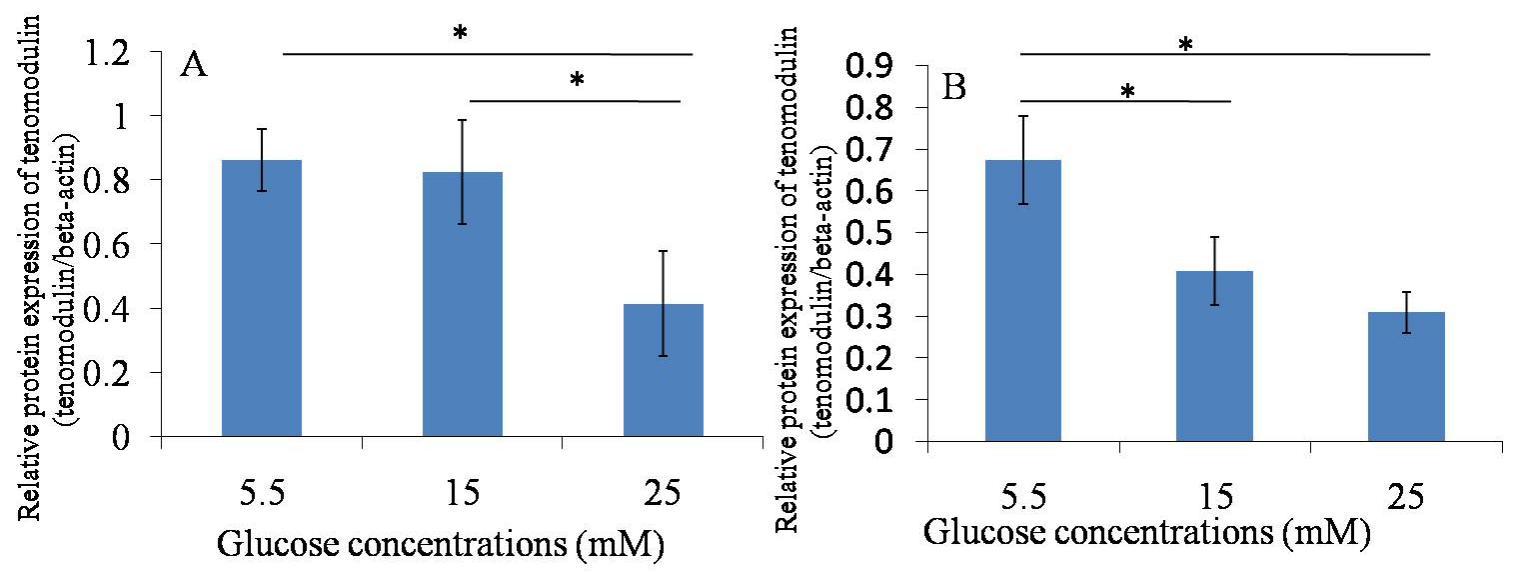
Western blotting ----Tnmd **A**. 24h after glucose induction; **B**. 48h after glucose induction; The protein expression of Tnmd was decreased in treatment with high glucose concentrations (15mM and 25mM) at 24 hours and 48 hours, compared with treatment with 5.5 mM glucose concentration. *P ≤ 0.05.

**Figure 7:**
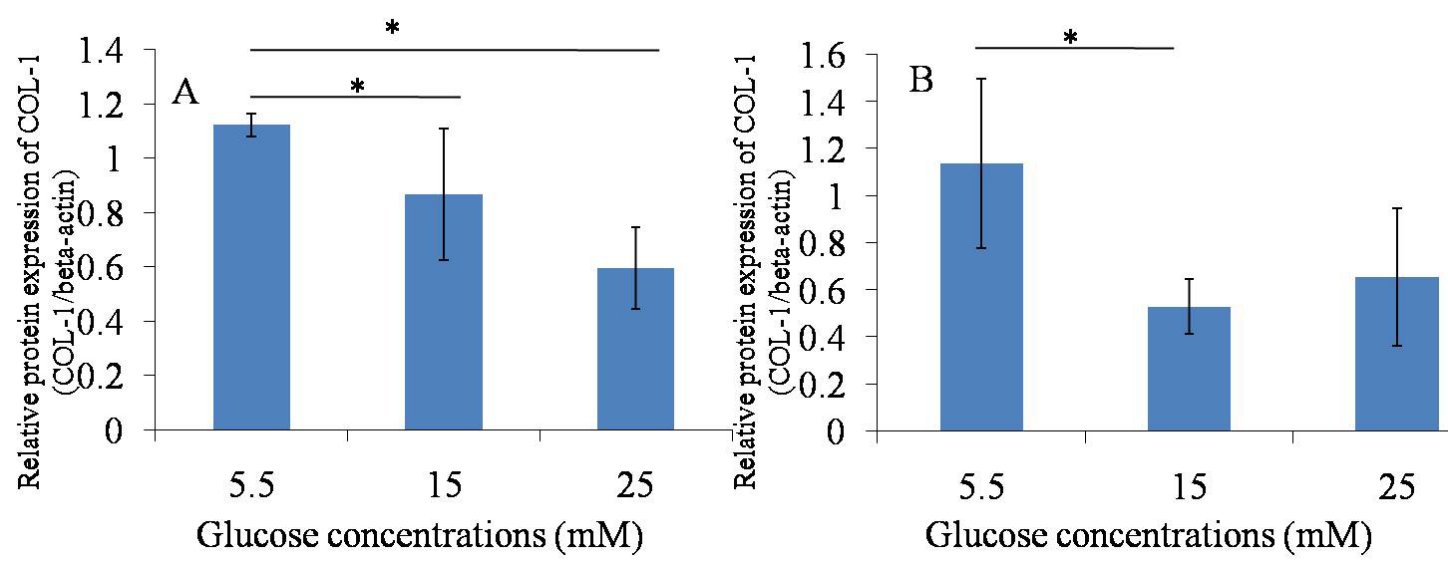
Western blotting ---- Col-1 **A**. 24h after glucose induction; **B**. 48h after glucose induction; The protein expression of Col-1 was decreased after the high glucose treatment (15mM and 25mM) for 24 hours and 48 hours compared with that in 5.5mM glucose group. *P ≤ 0.05.

The protein expression of Tnmd was lower in treatment with high glucose concentrations (15mM and 25mM) at 24 hours and 48 hours, compared with treatment with 5.5 mM glucose concentration. The expression of Col-1 was similar to Tnmd. The protein expression of Col-1 in TDSCs was decreased after the high glucose treatment (15mM and 25mM) for 24 hours and 48 hours compared with that in 5.5mM glucose group. (Figures 5, 6, 7).

## DISCUSSION

**Figure 8:**
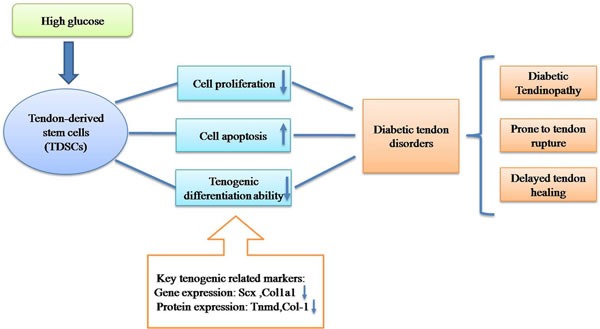
Hypothetical model of pathogenesis of diabetic tendon disorders

In this study, we found that high glucose could inhibit cell proliferation and induce cell apoptosis of TDSCs *in vitro*. The tenogenic differentiation ability of TDSCs was decreased in the initial stage (incubation time for 24h and 48h), *in vitro*. All these findings proved our hypothesis and accounted for the potential pathogenesis of diabetic tendon disorders including diabetic tendinopathy, tendon rupture and impaired tendon healing (Figure 8).

Our experiment was designed to simulate the normal blood glucose environment in non-diabetics and the hyperglycemic environment in diabetics *in vitro*. In healthy individuals, blood glucose is tightly sustained from 4mM to 7mM. In diabetics, levels can rise appreciably higher. Serious diabetic complications can arise when levels exceed 13 mM, and concentrations of up to 33 mM are often dangerous. Thomas et al. demonstrated that the glucose concentration in subcutaneous tissue closely resembled that in plasma [22]. As we know, healthy tendon does not have intrinsic blood supply and is separated with the surrounding muscle by tendon sheath. Our previous study also showed the cellularity and vascularity were insignificant in the healthy patellar tendons, however, hypercellularity and hypervascularity were observed in patellar tendinopathy samples [23]. The normally hypovascularized tendon tissue becomes hypervascularized during the procedure of degenerative disease [24]. The Achilles tendons in diabetic rats showed vascular feedbacks uncommon to healthy tendons, such as the increase in vascular density and vascular hyperplasia [25]. Our unpublished data also showed vascularization in tendons of diabetic animal model. Furthermore, there has been no report on the glucose concentration in tendon tissue in the normal population or in diabetic patients. Therefore, the glucose concentrations (5.5mM, 15mM and 25 mM) were used to simulate the normal and diabetic environments according to Thomas's research.

High blood glucose is the key pathogenic inducer that leads to diabetic complications including musculoskeletal disorders. The adverse effects of high glucose on various mesenchymal stem cells have been known for a long time. Increasing evidence indicates that high glucose inhibits the proliferation, migration and angiogenic ability of bone marrow-derived progenitor cells *in vitro*, and alters the regenerative potential of mesenchymal stem cells [26, 27]. Moreover, researchers demonstrate that high glucose inhibits the proliferation and mineralization of periodontal ligament stem cells *in vitro* [28]. Glucose overdose is a potential pathogenic factor of cytotoxic, genotoxic, and apoptotic effects on tumors cells [29].

This is the first study, to our knowledge, to discuss the effects of different glucose levels on TDSCs *in vitro*. In the present study, we demonstrated a decrease in the proliferative capacity of TDSCs in high glucose concentration level. Our results are similar to the previous reports mentioned above. We also found that TDSCs cultured in high glucose medium (15mM, 25mM) for 48h led to a significant increase in apoptosis. Similar results were found in the neural stem cells followed by high glucose intervention *in vitro* [30]. After injury, TDSCs would proliferate and differentiate into tenocytes in normal tendon healing process [15]. The proliferative potential and viability of TDSCs play a vital role in maintaining the physiological function of tendon. The reduced proliferation ability of TDSCs, coupled with increased apoptosis might reduce the pool of TDSCs for tendon repair in diabetic patients.

There are some key factors playing important roles in tendon differentiation. Tnmd is a tendon-specific marker known to be important for TDSCs tenogenic differentiation [8]. Scx is also a marker of the tenocyte lineage. Scx itself is a transcription factor, which directly regulates expression of col1a1 and col1a2 [31]. Furthermore, Shukunami, C., et al. found that Tnmd was a marker of tendon formation and Scx positively regulated Tnmd expression in a tendon cell lineage-dependent manner [32]. Tendon extracellular matrix mainly containing collagen type I, shows a highly organized parallel structure around the tenocytes. The synthesis of collagen type I is crucial to maintain tendon structure, for collagen type I is the main ingredient of tendons [33]. Mohawk homeobox (MKX) has been demonstrated as a tendon specific transcription factor. Previous studies have shown that Scx is essential for the initiation of tendon differentiation, whereas Mkx plays a vital role in tendon maturation [34]. In this study, we chose Scx instead of Mkx to see the ability of tendon differentiation. Tenascin C (TN-C) is expressed in the extracellular matrix of different cell types during development, disease or injury. Expression of TN-C varies from childhood to adulthood. TN-C is highly expressed during embryogenesis or in developing tendons, bone and cartilage while in developed organs, expression is absent or in a trace expression [35]. So, we believe that TN-C is a more appropriate maker for the stemness of TDSCs. Therefore, in this study, we investigated the mRNA expression of Scx and Col1a1, the protein expression of Tnmd and collagen I in order to compare tendon-related markers expression of TDSCs under different glucose concentrations *in vitro*. Our results showed that high glucose concentrations (15mM and 25mM) suppressed the gene expression of tenodon-related makers (Scx and Col1a1) and the protein expression of tendon-related markers (Tnmd and Col-1) after 24h and 48h culture. Because of this, the suppression of tendon-related markers of TDSCs may suggest that tenogenic differentiation ability of TDSCs is impaired in diabetic tendons. Exposure to high glucose environment leads to impaired tendon healing and making the tendon vulnerable to rupture in the initial stage. The findings are consistent with changes, such as disordered collagen fibers, reduced biomechanical properties, impaired tendon healing ability and decreased proteoglycan levels observed in injured diabetic tendons [17, 19, 36].

Actually, the molecular mechanisms of high glucose affecting stem cell proliferation, apoptosis and differentiation still remains unclear. In this study, our findings suggested that high glucose altered the fate of TDSCs *in vitro*. Snedeker JG illustrated that advanced glycation end-products (AGEs) pathologically disrupted tendon homeostasis and damaged tendon repair process [37]. The uncontrolled reactions between glucose and the extracellular matrix, excessive accumulation of AGEs, the altered inflammatory responses, neo-vascularization and dysregulation of TDSCs differentiation might account for mechanisms of the effects of high glucose on TDSCs. The underlying molecular mechanisms need further investigated.

There are also some limitations in this study. This experiment was an *in vitro* study, and incubation of TDSCs with high concentrations of glucose *in vitro* may not mimic the *in vivo* conditions of hyperglycemia in the patients. Therefore, further animal studies should be done to certify the effects of high glucose on TDSCs *in vivo*. Meanwhile, whether the glucose concentrations used in this study are the most appropriate need to be further verified. Moreover, the expression of tendon-related markers were showed a downward trend in this study. With the extension of incubation time in high glucose environment, whether TDSCs are activated again after injury and then self-healing need to do further research.

## CONCLUSIONS

High glucose could inhibit proliferation, induce cell apoptosis and suppress the tendon-related markers expression of TDSCs *in vitro*. These results may partly explain the pathological and molecular mechanisms of the pathogenesis of diabetic tendon disorders.

## MATERIALS AND METHODS

### Isolation and culture of rat TDSCs

All experiments were approved by the Animal Research Ethics Committee of Southeast University. The procedures for the isolation of TDSCs from rat patellar tendon have been well-established [10]. Briefly, the mid-substance of patellar tendons were excised from rats overdosed with 2.5% sodium phenobarbital. Care was taken that only the mid-substance of the patellar tendon tissue, but not the tissue at the bone-tendon junction, was collected. Peritendinous connective tissue was carefully removed and the samples were stored in sterile phosphate-buffered saline (PBS). The tissues were minced, digested with type I collagenase (3 mg/mL; Sigma-Aldrich), and passed through a 70μm cell strainer (Becton Dickinson, Franklin Lakes, NJ) to yield a single-cell suspension. The released cells were washed in PBS and resuspended in a complete culture medium [Dulbecco’s Modified Eagle Medium (DMEM, low glucose formulation, glucose concentration: 5.5mM), 10% fetal bovine serum, 100 U/mL penicillin, 100mg/mL streptomycin] (all from Invitrogen Corporation, Carlsbad, CA). The isolated nucleated cells were plated at an optimal low cell density (50 nucleated cells/cm^2^) for the isolation of stem cells and cultured at 37°C, 5%CO_2_ to form colonies. At day 2, the cells were washed with phosphate buffered saline (PBS) to remove the nonadherent cells. At day 7, they were trypsinized and mixed together as passage0 (P0). Cells from P2 to P5 were used for all experiments. Medium was changed every 3 days. The clonogenicity and multi-lineage differentiation potential of these cells were confirmed before being used for the experiments in this study using standard assays as described previously [10]. We added D-Glucose (from Sigma-Aldrich, USA) with different weight to DMEM in order to make different concentrations of glucose in the cell culture medium.

### MTT (3- [4,5-Dimethylthiazol-2-yl]-2,5-diphenyltetrazolium bromide; Thiazolyl blue) assay

The MTT assay was used to measure cell survival and proliferation. TDSCs were plated into 96-well culture plates at an optimal density of 3 × 10^3^cells/well in 200 μL complete culture medium. After 24 hours culture, the complete culture medium was aspirated and discarded from each well, then the cells were treated with medium containing different concentrations of glucose (5.5mM, 15mM and 25mM). The cells were observed under a microscope and then the MTT assay was performed at day1, day3 and day5 after high glucose culture. DMEM, containing 5 mg/mL MTT (Sigma-Aldrich, USA), was added to each well and the plate was incubated at 37°C for 4 hours. Then, the MTT solution was removed and 150μL of dimethyl sulfoxide [DMSO (Sigma-Aldrich, USA)] was added to each well. After the crystals were dissolved by mixing with micro-pipette, the colorless DMSO turned purple. Subsequently, the absorbance of each well was immediately measured with an enzyme-linked immunosorbant assay reader (model: mk3; Thermo Scientific, USA) at 570 nm. These experiments were performed in triplicate.

### Apoptosis assay

A propidium iodide (PI) and annexin V-FITC-flow cytometry assay (BD Pharmingen) was used to detect the apoptosis rate in the cells after the induction of different glucose concentrations. Briefly, 5×10^4^cells per well were cultured in 6-well plates in the complete culture medium for 24 hours. Then, the complete culture medium was aspirated and discarded from each well, cells were treated with medium containing different concentrations of glucose (5.5mM, 15mM and 25mM) for 24 hours and 48 hours. Adherent cells were detached with 0.25% trypsin without EDTA in 1×PBS. Cells were harvested in PBS and centrifuged at 2000 rpm for 5 minutes. Then the cells were washed in PBS, re-suspended in binding buffer (BD Pharmingen), and stained with FITC-conjugated annexin V and propidium iodide (Pharmingen, Becton Dickinson Co., San Diego, CA, USA). After staining, the cells were incubated for 15 minutes in the dark at room temperature. Cells were analysed by flow cytometry (FACS Calibar; Becton-Dickinson) using Cell Quest software.

### Quantitative real-time reverse transcription-polymerase chain reaction (qRT-PCR)

qRT-PCR was performed as previously described [38]. After the culture of different concentrations of glucose medium (5.5mM, 15mM and 25mM) for 24h and 48h, cells were harvested and homogenized for RNA extraction with the Rneasy mini kit (Qiagen GmbH, Hilden, Germany). The mRNA was reverse transcribed to cDNA by the First-Strand cDNA kit (Promega, Madison, WI). 1μL of total cDNA of each sample was amplified in the final volume of 20μL of reaction mixture containing Power SYBR Green PCR Master Mix (Invitrogen Corporation, Carlsbad, CA) and specific primers for Scx and Col1a1 using the ABI StepOne Plus system (all from Applied Biosystems, CA, USA) (Table 1). Cycling conditions were denaturation at 95°C for 10 min, 45 cycles at 95°C for 20 s, optimal annealing temperature (Table 1) for 20 s, 72°C for 30 s, and finally at 60°C-95°C with a heating rate of 0.1°C/s. The expression of the target gene was normalized to that of the β-actin gene. Relative gene expression was calculated using the 2^-ΔCT^ formula.

**Table 1:** Primer sequences and condition for qRT-PCR.

Gene	Primer nucleotide sequence	Product size (bp)	Annealing temperature (°C)	Accession no.
β-actin	5'-ATCGTGGGCCGCCCTAGGCA-3’ (forward) 5'-TGGCCTTAGGGTTCAGAGGGG-3' (reverse)	243	52	NM_031144
Scleraxis	5’-AACACGGCCTTCACTGCGCTG-3’ (forward) 5’-CAGTAGCACGTTGCCCAGGTG-3’ (reverse)	102	58	NM_001130508.1
Col1a1	5'- CATCGGTGGTACTAAC-3'(forward) 5'- CTGGATCATATTGCACA-3'(reverse)	238	55	NM_053356.1

### Western blotting

After the culture of different concentrations of glucose medium (5.5mM, 15mM and 25mM) for 24h and 48h, the cells were lysed, centrifuged, and the supernatant was then collected for measurement of protein concentration by BCA protein assay (Thermo Scientific, Rockford, IL, USA ). 50μg of protein was denatured, fractionated by electrophoresis on 10% (Tnmd) or 8% (Col-1) (w/v) SDS-PAGE and electrophoretically transferred to a PVDF membrane (Millipore, Billerica, MA). The blots were blocked with 5% (w/v) nonfat dry milk in TBST solution [25 mM Trizma base (3.025g), 125 mM NaCl (7.3g) and 1 ml Tween-20, pH 7.6], incubated with primary antibody against Tnmd (1:1000), Col-1 (1:1000) (all from Abcam, Cambridge, UK), followed by horseradish peroxidase-conjugated secondary antibody (1:3000; Santa Cruz Biotechnology, Santa Cruz , CA , USA). Immunoreactive bands were detected by ECL reagents (Pierce Biotechnology Inc., Rockford, IL). The membranes were stripped with Restore Western blot stripping buffer (Pierce Biotechnology Inc., Rockford, IL) and reprobed with β-actin antibody (1:3000) (R&D Systems, Inc., Minneapolis, MN) as a house-keeping control. Semiquantitative image analyses of protein expression were performed using the Image J Software (Wayne Rasband, National Institutes of Health, USA), and the mean expression level of the target protein relative to β-actin was presented.

### Data analysis

All data are presented in mean ± standard deviation (SD). For statistical analysis of the data in this assay, comparison of more than two groups was done using Kruskal-Wallis test followed by post hoc comparison with control group using Mann-Whitney U-test. Differences between groups were measured by the Mann-Whitney U-test. All the data analysis was done using SPSS analysis software (SPSS, Inc., Chicago, IL; version 18.0). P≤0.05 was regarded as statistically significant difference.

## SUPPLEMENTARY MATERIALS TABLES



## Abbreviations

TDSCs: tendon-derived stem cells; Tnmd: tenomodulin; Scx: scleraxis; Col-1: collagen I; col1a1: collagen I alpha 1 chain; DMEM: Dulbecco's Modified Eagle Medium.
