# Supplementary material for: The effects of high glucose on tendon-derived stem cells: implications of the pathogenesis of diabetic tendon disorders
Source: Oncotarget. 2017 Feb 16;8(11):17518–28. doi: 10.18632/oncotarget.15418 (PMC5392267; doi:10.18632/oncotarget.15418)
Supplement: Supplementary file 1 [file oncotarget-08-17518-s001.pdf]

# The effects of high glucose on tendon-derived stem cells: implications of the pathogenesis of diabetic tendon disorders

## Supplementary Material

**Supplementary Table 1: Primer sequences and condition for qRT-PCR**

| Gene | Primer nucleotide sequence | Product size (bp) | Annealing temperature (℃) | Accession no. |
|---|---|---|---|---|
| β-actin | 5'-ATC GTG GGC CGC CCT AGG CA-3' (forward)<br>5'-TGG CCT TAG GGT TCA GAG GGG-3' (reverse) | 243 | 52 | NM_031144 |
| Scleraxis | 5'-AACACGGCCTTCACTGCGCTG-3' (forward)<br>5'-CAGTAGCACGTTGCCCAGGTG-3' (reverse) | 102 | 58 | NM_00113050 8.1 |
| Col1a1 | 5'- CATCGGTGGTACTAAC-3'(forward)<br>5'- CTGGATCATATTGCACA-3'(reverse) | 238 | 55 | NM_053356.1 |
